# Gender Differences in Mortality Risk After Driving Cessation Among Older Men and Women: a Mediation Analysis

**DOI:** 10.1093/geroni/igaa057.956

**Published:** 2020-12-16

**Authors:** Jiao Yu, Eva Kahana, Boaz Kahana, Yuhan Zhang

**Affiliations:** 1 Case Western Reserve University, Cleveland, Ohio, United States; 2 Cleveland State University, Cleveland, Ohio, United States

## Abstract

Driving is the most important personal transportation mode in the US for maintaining mobility. Previous studies of older adults who stop driving have identified several health risks associated with driving cessation, including less access to health care, increased dependency, social isolation, and elevated risk of mortality. The purpose of this analysis was to examine driving status as a predictor of mortality among community-dwelling older men and older women. Data were drawn from a prospective panel study of successful aging project of 1000 older adults (mean age = 80). Participants’ driving status was measured at baseline and mortality rates were observed across the subsequent 10 years. Extended Cox proportional hazard model indicated a 76% (p<0.001) significantly higher mortality risk for non-drivers versus drivers. This relationship was mediated by health conditions and functional status for both older men and older women. Among older men, health status fully mediates the association between driving cessation and mortality risk. A partial mediation effect of health status on the association between driving cessation and mortality risk was found among older women. Older women who stopped driving faced 56% (p>0.01) higher relative mortality risk than their driver counterparts. Social and cultural issues such as gender stereotype, autonomy, and social connection with their implication for driving may explain existing gender differences among older adults.

